# Prevention and management of intra‐operative complications in maxillary sinus augmentation: A review

**DOI:** 10.1111/cid.13397

**Published:** 2024-10-08

**Authors:** Pascal Valentini, Claudio Stacchi

**Affiliations:** ^1^ Institute of Health, Department of Implant Surgery, Tattone Hospital University of Corsica Pasquale Paoli Corte France; ^2^ Department of Medical, Surgical and Health Sciences University of Trieste Trieste Italy

**Keywords:** intraoperative complications, lateral approach, maxillary sinus augmentation, membrane perforation, transcrestal approach, vascular damage

## Abstract

Maxillary sinus floor elevation is usually performed in two different ways: the lateral approach involves the creation of a bony window on the maxillary sinus lateral wall, providing direct access to the sinus cavity for membrane elevation and subsequent graft placement, and the transcrestal approach is considered less invasive. The aim of this article is to describe, based on the literature, how to anticipate, avoid, and manage the intraoperative complications that can occur with both approaches. For both approaches, the most common complication is the sinus membrane perforation. For the lateral approach, an average frequency ranging from 15.7% to 23.1% is reported, but because of the better visibility, their management will be easier compared to the transcrestal approach. Mean perforation rate reported for the transcrestal approach is lower (3.1%–6.4%), but it should be noted that a significant number of perforations cannot be detected and managed given the blind nature of this technique. Anatomical parameters such as sinus width and buccal wall thickness may be a risk factor for one approach and not the other. As it is impossible to assess the resistance of the Schneiderian membrane, the transcrestal approach is more likely to lead to infectious complications in the event of perforation. Others, such as the risk of vascular damage, are encountered only with the lateral approach, which can be prevented easily by dissecting the alveolo‐antral artery. For both approaches, prevention is essential and consists in analyzing the anatomy, mastering the surgical technique, and collaborating with the ENT to manage the essentially infectious consequences of intraoperative complications.

**Summary Box**

**What is known**

- Intraoperative complications may lead to postoperative infections.
- Transcrestal approach is less invasive compared to the lateral approach.

**What this article adds**

- Lateral approach allows better management of sinus membrane perforations.
- The transcrestal approach, being a blind technique, requires special care in preoperative planning to avoid complications.

## 1 INTRODUCTION

Maxillary sinus floor elevation, also known as sinus lift, is a commonly performed procedure in oral implantology to increase the available bone height in the posterior maxilla, facilitating successful implant placement. The two most common surgical approaches for maxillary sinus lift are the lateral approach (the first to be proposed over 40 years ago)^1^ and the transcrestal approach,^2^ aimed at limiting the invasiveness of the conventional procedure while seeking to achieve similar clinical results.

The lateral approach involves the creation of a bony window on the maxillary sinus lateral wall, providing direct access to the sinus cavity for membrane elevation and subsequent graft placement. The main advantage of this technique is that the surgeon has the opportunity to directly control the most delicate part of the procedure, namely, the detachment and elevation of the sinus membrane.^3^ This allows for optimal lifting of the Schneiderian membrane from the bony walls of the sinus regardless of the size of the cavity and the creation of an adequate sub‐sinus space, which allows for the placement of the graft even in contact with the medial wall of the sinus, optimizing new bone formation.^4^, ^5^

Transcrestal sinus floor elevation, also known as the osteotome technique, is a minimally invasive approach to sinus augmentation.^2^ Unlike the lateral approach, transcrestal sinus floor elevation entails making a minimal antrostomy on the alveolar crest, performed using osteotomes, piezoelectric inserts, or specially designed burs.^6^, ^7^, ^8^ This opening is frequently utilized for implant placement following the elevation of the sinus membrane and grafting procedure. The main limitation of the transcrestal approach is that membrane elevation occurs indirectly, lifted hydrodynamically using saline solution or by granular or injectable biomaterial (paste or gel).^9^, ^10^ The surgeon lacks direct control over the direction and extent of membrane elevation: in transcrestal sinus floor elevation, predictable exposure of both lateral and medial bone walls occurs primarily in narrow maxillary sinuses.^11^

While advancements in biological understanding and surgical techniques have improved the predictability and success rate of maxillary sinus augmentation, intraoperative complications remain a concern for clinicians.^12^ In both lateral and transcrestal approaches, they can range from minor issues to more severe events, potentially leading to surgical failure or increased postoperative morbidity. Preventing and managing possible intraoperative complications is a crucial point for improving patient safety and treatment success. Clinicians should anticipate and address potential risks through strategic planning and execution and, at the same time, be aware that effective management of complications is essential for minimizing adverse effects and enhancing surgical procedure success.^12^, ^13^

Throughout this review, we will examine various aspects of intraoperative complications in both lateral and transcrestal maxillary sinus augmentation, including anatomical considerations, surgical techniques, graft materials, and patient‐related factors. By addressing each of these components comprehensively, we aim to offer practical guidance and recommendations that can inform clinical decision‐making and, ultimately, improve clinical outcomes.

## 2 INTRAOPERATIVE COMPLICATIONS

### 2.1 Sinus membrane perforation

Sinus membrane perforation is the most frequent complication in maxillary sinus augmentation with lateral approach, with an average frequency ranging from 15.7% to 23.1%,^12^, ^13^ but with significant variations across individual studies (0%–60%)^13^ depending on the surgeon's experience, surgical technique, anatomical conditions, and patient‐related factors.^14^, ^15^, ^16^, ^17^ Mean perforation rate reported for the transcrestal approach is lower (3.1%–6.4%),^16^, ^18^, ^19^, ^20^ but it should be noted that a significant number of perforations may not have been detected, given the blind nature of this technique. Indeed, when endoscopy was employed to confirm sinus membrane perforation during transcrestal sinus floor elevation in cadaver specimens, this rate increased significantly to 40%.^21^

Perforation of the sinus membrane can result in dissemination of grafting material into the sinus cavity, potentially compromising the patency of the ostiomeatal complex and triggering local inflammation often leading to postoperative sinusitis.^22^, ^23^, ^24^ Recognizing and addressing risk factors associated with membrane damage is essential to achieving successful outcomes in sinus augmentation procedures.^25^

#### 2.1.1 Risk factors and prevention

##### Antrostomy technique

###### Lateral approach

Even if some clinical studies reported no difference in membrane perforation risk between rotary and piezoelectric instruments,^26^, ^27^, ^28^ systematic reviews and meta‐analyses concluded that membrane perforations during lateral sinus augmentation may be significantly reduced applying piezoelectric devices.^14^, ^29^ However, in studies comparing the use of piezoelectric bone surgery for creating a lateral access to the sinus, outcomes varied depending on the surgical approach. When bone window outlining and reflection into the sinus were performed, perforation prevalence was 17.6%, similar to rotary instruments.^14^ However, the prevalence decreased significantly to 4.7% when ultrasonic instruments were used to thin the lateral wall before opening the window.^30^ This approach allows, especially when the lateral wall of the sinus is thick, to have better surgical control, to perceive the proximity to the membrane thanks to the change in color (the thinned area becomes darker because the sinus cavity is visible through it in transparency), and to clearly identify the position of Underwood septa, if present. Overall, within the limitations of the available studies, it appeared that thinning the lateral wall with ultrasonic instruments or bone scrapers reduces the incidence of accidental Schneiderian membrane perforations during antrostomy.

###### Transcrestal approach

There are no studies in the literature directly comparing the various techniques used to create crestal access to the maxillary sinus (manual or electric‐driven osteotomes, piezosurgery, various burs designed for this specific application) with regard to perforation prevention. In the absence of sufficient evidence, it is suggested to apply the technique in which the operator is experienced and has achieved a good learning curve to limit the risk of perforation at this stage of the surgical protocol.

#### 2.1.2 Anatomy

##### Membrane thickness

The thickness of the sinus membrane has long been a subject of debate. While some consider a thick membrane as indicative of resilience and view a thin membrane as fragile,^31^, ^32^ studies on cadaver specimens^33^ have shown that membrane thickness does not consistently correlate with resistance to tearing. Similarly, it has been demonstrated that sinus membranes measuring 1–1.5 mm in thickness exhibit greater resilience compared to those that are thinner or thicker.^34^

Since the literature reports contradictory information, it is difficult to predict the risk of perforation based solely on the thickness of the membrane observed on CBCT, which is often overestimated.^33^

##### Gingival phenotype

This parameter may also be considered during pre‐surgical planning to aid in assessing the risk of membrane perforation.^23^ It has been noted that a thick gingival phenotype is correlated with a thick sinus membrane, and vice versa.^35^, ^36^ However, as discussed earlier, establishing a direct correlation between phenotype and the risk of perforation presents challenges.

##### Sinus width

The width of the sinus cavity is an important factor to consider in the risk assessment for Schneiderian membrane perforations.^37^, ^38^, ^39^ This parameter can be assessed by measuring the angle between the buccal and palatal walls on CBCT cross‐section images. Normally, this angle is narrower in the most mesial area of the cavity as we approach the anterior wall, while it tends to become wider as we move in the distal direction.

**FIGURE 1:**
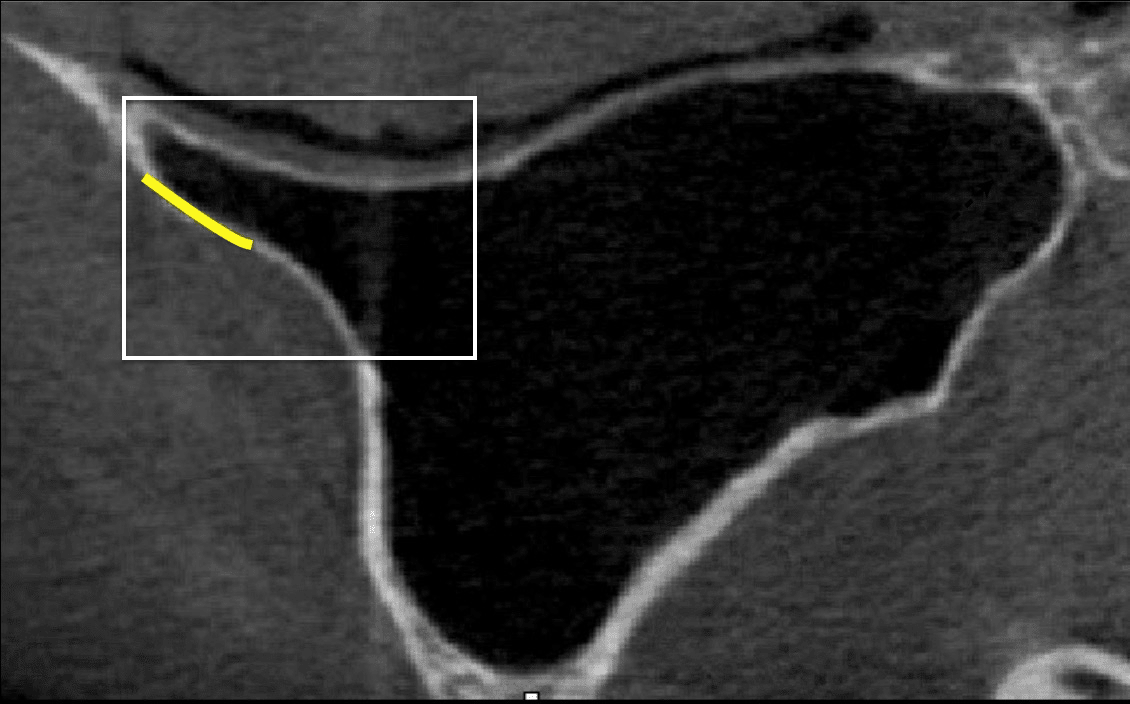
The window (yellow line) must be as anterior as possible (white rectangle).

In the lateral approach, when this angle is <30°, the prevalence of perforation exceeds 60%, decreasing as the angle becomes wider.^37^ This could be due to the difficulty in finding the correct cleavage plane for membrane detachment and therefore using manual instruments appropriately in such a narrow space. Therefore, the anterior part of the sinus cavity, being a narrow zone, represents a high‐risk area when performing lateral approach^12^, ^13^ (Figure 1). Consequently, the surgical window should be positioned as anteriorly as possible to allow for direct visualization and dissection of the membrane in the most critical area.^3^

Interestingly, the width of the sinus cavity seems to have completely different effects on influencing the risk of membrane perforation in the transcrestal approach. A recent multicenter study conducted on 430 patients showed a significant correlation between bucco‐palatal sinus width and membrane perforation, with an extremely low perforation rate observed in narrow sinuses and a much higher incidence in wide sinuses.^40^ This finding is a clinical confirmation of a principle described by Pommer et al. (2009),^32^ demonstrating that the force needed for membrane detachment during transcrestal sinus elevation increases with the size of the elevated area. When this force surpasses the sinus membrane elastic properties, perforation can occur. In narrow sinuses, where the elevated area is smaller compared to wide sinuses, higher elevation heights can typically be achieved before membrane tearing occurs. It is noteworthy that this situation is the exact opposite of what happens in sinus floor elevation with a lateral approach, creating an interesting complementarity between the two surgical techniques.

##### Palato‐nasal recess

The palato‐nasal recess, located between the roof of the hard palate and the lateral wall of the nasal cavity, can be found at various heights on the medial wall of the maxillary sinus.^41^ The average height of this recess gradually decreases from the premolar to the molar sites. Its position and angulation can impact the level of difficulty encountered during membrane elevation on the medial wall, in both lateral and transcrestal approaches: the sharper this angle, the higher the risk of perforation. Taking into account both the location and angulation, ~15% of premolar sites may exhibit an acute‐angled palato‐nasal recess, complicating membrane elevation. In contrast, this condition is observed in only about 2% of molar sites within the surgical area of sinus floor elevation.^22^, ^41^

##### Thickness of the buccal wall

The thickness of the lateral wall plays a role in the risk of perforation only during lateral approach.^39^, ^42^, ^43^

**FIGURE 2:**
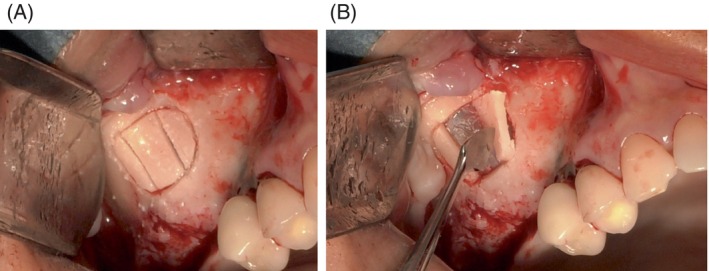
(A) The window is divided into three small pieces. (B) All pieces are removed successively.

As said previously, the ultrasonic erosion of the lateral wall is the safest approach to avoid membrane perforation.^14^ However, if the lateral bone wall is thick, erosion can be time‐consuming, and complete removal may be preferred.^44^, ^45^ Nevertheless, complete removal carries a risk of perforation due to limited surgical control, especially if the membrane is strongly attached and in the presence of Underwood septa. To prevent tearing, the bony lid can be divided into several pieces and detached one by one (Figure 2a,b).^46^ This approach reduces the pull‐out force but is contraindicated if there is an intraosseous passage of the alveolo‐antral artery.

Clearly, the thickness of the buccal wall has no influence on the risk of sinus membrane perforation during a transcrestal approach.

##### Septa

The identification of Underwood septa underscores the importance of thorough 3D preoperative imaging to meticulously assess the internal sinus anatomy, given its significant impact on surgical approach.^47^ The anticipated risk of perforation is lower with medio‐lateral septa compared to antero‐posterior septa,^28^ necessitating the implementation of a specific technique tailored to the anatomy.^48^ As an example, when the sinus is divided into three different cavities by two full medio‐lateral septa, it is preferable to plan the creation of three windows.^49^ If encountering an antero‐posteriorly oriented septum, it should be removed using ultrasonic osteotomy at its base after elevating the membrane to expose the septum.^46^ Alternatively, it is possible to combine the buccal approach with a palatal window without the need to remove the septum. An innovative approach^50^ has been suggested when the sinus presents complex septa and sinus floor convolutions. This technique involves partially cutting and removing septa followed by mucosal elevation without graft placement. After a few months, the sinus mucosa thickened due to scarring, allows for more predictable membrane elevation.

**FIGURE 3:**
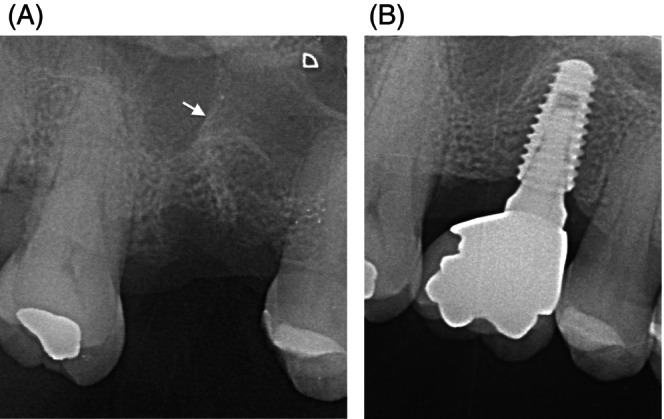
(A) The septum is in the center of the implant site (white arrow). (B) The graft and the implant are located mesially.

Also in the transcrestal approach, it is essential to precisely identify the position and orientation of Underwood septa to assess any potential impact on the regenerative procedure. If a medio‐lateral septum is present at the augmentation site, sinus crestal access and subsequent grafting should be planned either mesially or distally to it (Figure 3a,b). When the septum orientation is antero‐posterior, crestal osteotomy and graft insertion should be carried out medially or laterally to it. However, this last option is feasible only if the sinus cavity is wide enough in medio‐lateral direction to allow a proper implant placement at crestal level.

##### Residual bone height (RBH)

Regarding this parameter, due to conflicting evidence in the literature, reaching a definitive conclusion is challenging for the lateral approach.^23^, ^51^, ^52^ An exception may arise when the residual bone height (RBH) is <4 mm and implants are placed simultaneously. To reduce the risk of fracture of the residual bone crest due to implant insertion in undersized sites, the surgical window should be moved apically.^46^, ^53^ However, in such cases, the detachment of the membrane from the lower border of the window to the sinus floor will be performed blindly and may pose a higher risk of tearing, particularly in the presence of septa.

Regarding the transcrestal approach, the available evidence in the literature does not demonstrate a significant direct influence of residual bone crest height on the risk of membrane perforation.^54^ On the contrary, from a clinical perspective, a very low residual crestal height may improve surgical visibility, allowing for better control by the operator over membrane integrity and the initial stages of biomaterial insertion.

##### Location and type of edentulism

In the context of the lateral approach, a study^51^ indicates a higher incidence of perforations (41.2%) in premolar–molar edentulous areas compared to premolar sites (16.7%) and molar sites (26.2%). However, these findings somewhat conflict with the findings regarding the influence of sinus width on membrane perforation during lateral sinus augmentation.^37^ Managing a single missing tooth appears to present greater challenges.^55^

There is no evidence supporting direct associations between the risk of membrane perforation, location, and type of edentulism in transcrestal sinus lift procedures.

#### 2.1.3 Patient‐related factors

##### Surgical access

In the lateral approach, the surgical access can pose a risk due to the necessity of working perpendicularly to the lateral wall for maximum effectiveness. This requirement can be met more or less easily depending on the morphological biotype of the patient. Achieving this access can be more challenging in brachycephalic patients compared to dolichocephalic patients.^12^

In transcrestal sinus augmentation, membrane perforation risk increases in regions with a sloped sinus floor: in these cases, the critical moment is during the execution of the crestal access, both with osteotomes and specific burs. The instrument initially contacts the sinus membrane at the point where the remaining bone crest is at its lowest height. As the antrostomy procedure progresses, the instrument continues to actively engage with the membrane at this location, thereby increasing the risk of tears.^21^, ^54^

##### Smokers

Several clinical studies and meta‐analyses have demonstrated a strong association between the prevalence of membrane perforations during sinus floor elevation with lateral approach and smoking,^25^, ^51^, ^52^ although it was not possible yet to correlate risk with the number of cigarettes smoked per day.^56^

Although it seems reasonable to assume that membrane changes induced by smoking similarly affect the risk of perforation in both the lateral and transcrestal approaches, insufficient scientific evidence is currently available to confirm this hypothesis.

#### 2.1.4 How to diagnose a perforation?

Timely identification of perforations is critical for effective management. Although the Valsalva maneuver is commonly used, it has limitations and may not always be the most reliable method, especially showing a significant number of false negatives, particularly in transcrestal approaches.^57^ A simpler alternative involves injecting sterile saline solution into the sub‐antral space and observing if the patient feels fluid flowing into their nose, indicating a breach. The use of endoscopy is undoubtedly the most accurate and reliable method for detecting perforations in both lateral and transcrestal approaches, but this equipment is typically not readily available in routine clinical practice.

Given the challenge of accurately assessing sinus membrane resistance capacity, the use of a more comprehensive tool to evaluate the level of difficulty of the specific case, as proposed by Testori and colleagues, can be beneficial.^13^ Based on the score obtained from considering anatomical and patient‐related parameters, we can determine whether to adopt the lateral approach, which enables precise visualization and treatment of perforations.

The impact of perforations on new bone formation and implant success is still a subject of debate,^58^, ^59^ although recent meta‐analyses have indicated a negative effect of intraoperative membrane tearing on these outcomes.^16^, ^22^, ^23^

However, proper management of the complication and appropriate repair of the membrane tear are fundamental elements to ensure the success of the regenerative procedure.^52^, ^60^, ^61^

### 2.2 Management of membrane perforations

**FIGURE 4:**
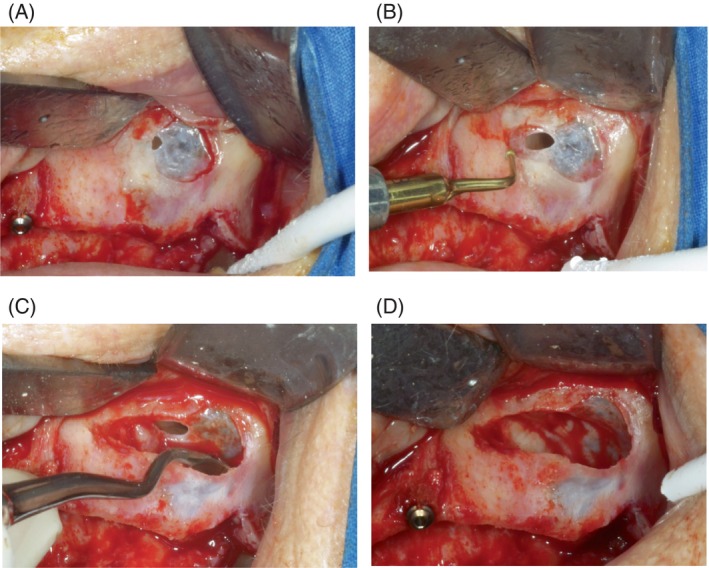
(A) The perforation is tangent to the osteotomy line. (B) The shape of the window is modified to see the perforation in its entirety. (C) The detachment is initiated opposite to the perforation. (D) The perforation size is reduced.

Perforations often occur during the creation of the lateral window, especially when the osteotomy line grazes the edge, making the breach partially hidden (Figure 4a). Adjusting the shape of the window to fully expose the perforation (Figure 4b) is an important initial step, as achieving optimal intraoperative visibility of the area is essential for a correct management of this complication.^46^

Starting the detachment of the membrane from the side opposite the perforation (Figure 4c) can exploit membrane elasticity to reduce and partially close the tear (Figure 4d).

The goal is to work on the membrane, continuing to detach it without causing an increase in the size of the perforation and achieving a secure seal to prevent graft material migration into the sinus cavity. Repair using a collagen membrane is the most commonly utilized method for fixing perforations and maintaining airtightness, with diverse techniques proposed across various studies.^61^, ^62^ However, even with collagen membrane repair, the integrity of the Schneiderian membrane and the potential for new bone formation could be compromised.^62^, ^63^, ^64^ This challenge arises from the difficulty in assessing membrane resistance to the pressure applied during graft condensation. To address this issue, it is important to compact the graft material against solid bone surfaces, avoiding direct pressure on the membrane to prevent re‐opening of the treated perforation or the creation of new tears.

**FIGURE 5:**
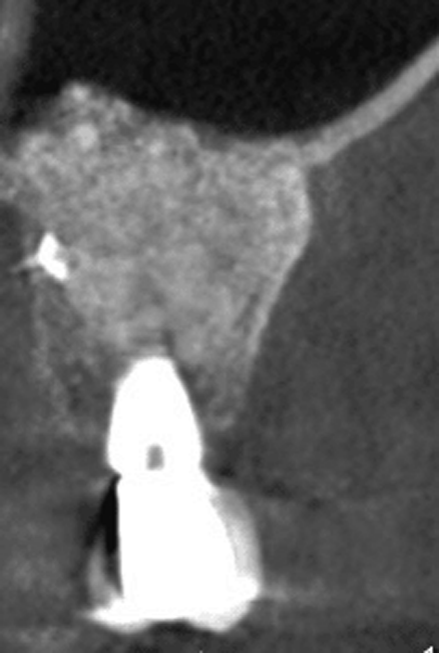
The membrane is pinned in the medial wall and the buccal wall.

**FIGURE 6:**
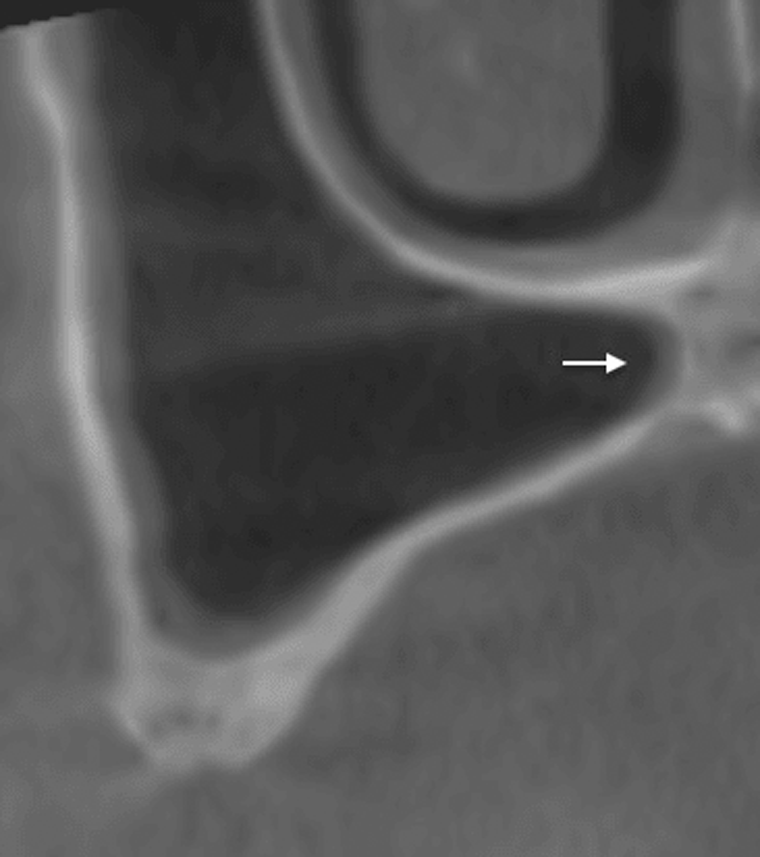
The palato‐nasal recess is acute (white arrow).

Previous reports showed a higher incidence of sinusitis (31.4%) in cases of membrane perforation, despite attempts to close the perforation with resorbable membranes,^52^ indicating that membrane stability during graft placement may not always be guaranteed.^65^ To address this issue, techniques such as the Loma Linda Pouch Technique^66^ involve the use of a large resorbable membrane that is folded into the sinus to fully enclose the graft material positioned at its center. However, during the membrane repair procedure, it is important not only to contain the graft material but also to preserve the blood and cellular supply to the grafted area, facilitating new bone formation. From a biological point of view, it is important to note that the collagen membrane used in the Loma Linda pouch technique completely isolates the graft material from the vascular and cellular supply originating from the sinus walls during the early phases of healing.^65^ An alternative that allows for stabilizing the membrane without hindering the biological processes necessary for new bone formation is the Tattone Technique.^67^ This approach involves shaping the membrane appropriately and then fixing it with titanium pins on the medial wall and, if necessary, on the buccal wall (Figure 5). This technique is particularly useful when the tear is located near the medial wall, a frequent occurrence in anatomies where an acute‐angled palato‐nasal recess is present (Figure 6).^22^, ^41^, ^46^

Another possibility proposed in the literature to manage membrane perforations is to suture them with 6.0 or 7.0 resorbable thread.^61^ This option may be valid only when dealing with thick membranes; attempting to suture a perforation on a thin membrane could be detrimental and could potentially widen the perforation.^68^

Another promising strategy to mitigate complications related to granule loss through perforation could involve using PRF membranes^69^, ^70^ to cover the perforations and as grafting material or without any grafting material,^71^ with reported implant survival rates of 100% and 98.7%, respectively.

In cases of large perforations, a preferable course of action may involve aborting the procedure and closing the flap, allowing for a waiting period of 4–8 weeks before resuming the procedure using a split thickness approach.^72^

In the transcrestal approach, Tavelli and colleagues (2020)^73^ suggested a classification guiding the clinical management of intraoperative perforations.

If the perforation occurs during the creation of the crestal antrostomy (Type 1), the clinician must assess whether it is possible to place a short implant, based on the height of the residual bone crest. If this is feasible, the implant should be inserted after protecting the sinus membrane with a collagen sponge, without placing any graft material. If the height of the residual alveolar crest does not allow for the placement of a short implant, it is suggested to proceed with creating a lateral window to manage the perforation. The same treatment approach should be adopted in cases of perforations occurring during membrane elevation or graft insertion (Type 2).

If perforation occurs during implant placement (Type 3), characterized by a diffuse radiopacity appearance on clinical examination, the patient should be closely monitored with frequent follow‐ups. If symptoms of sinusitis develop (such as chronic nasal drainage, pain, or the presence of mucosal fistula), the patient should be treated with medical therapy, and, in consultation with the otolaryngologist, partial or complete removal of the graft should be considered.

#### 2.2.1 Delamination

**FIGURE 7:**
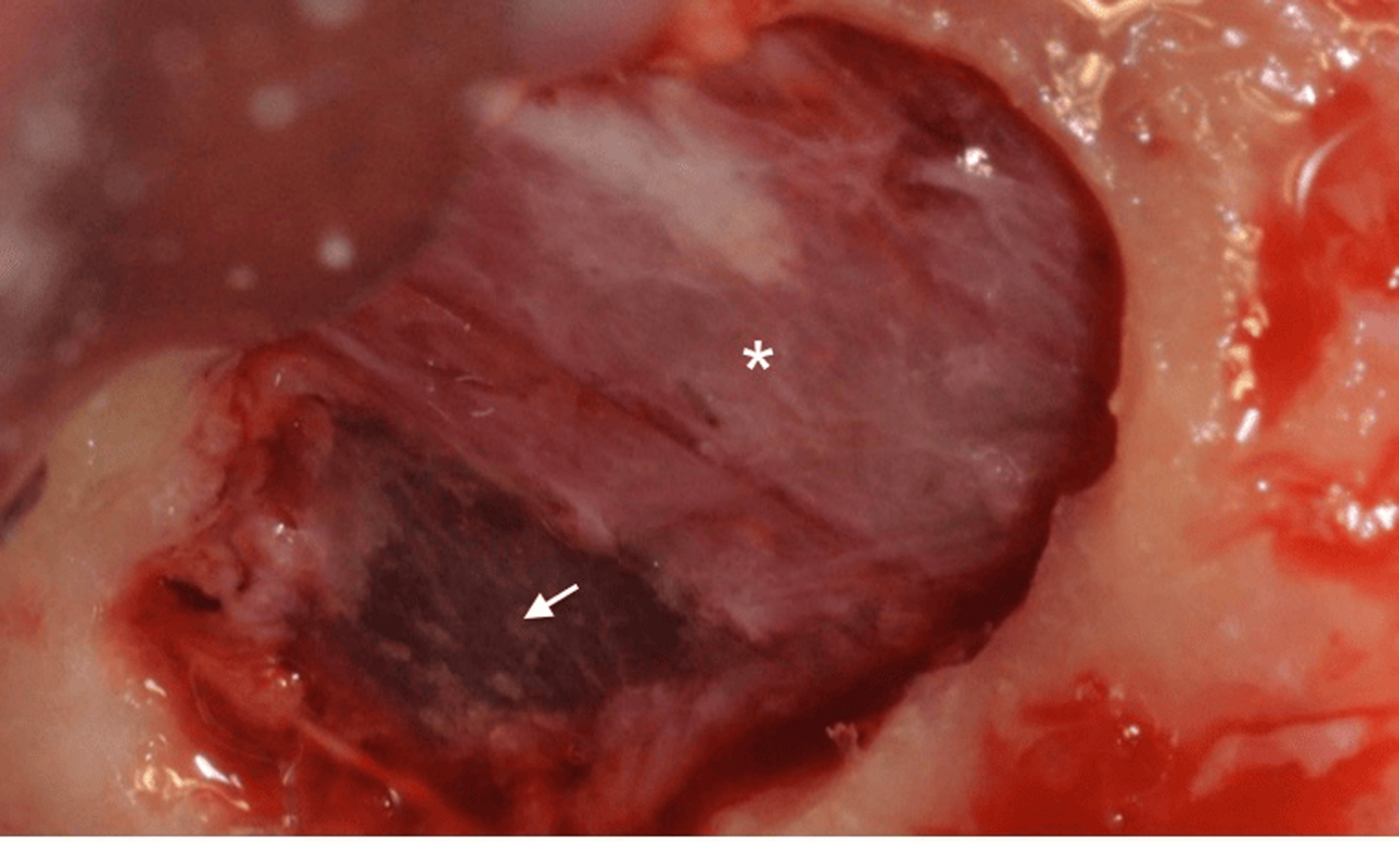
The intact periosteal part (white star) surrounding the delamination (white arrow).

Partial damage to the Schneiderian membrane may occur either during the lateral antrostomy or while detaching and elevating the membrane, leading to a tear in its periosteal component (Figure 7). Such damage could likely transform in a full perforation during the continuation of the surgical procedure or postoperatively, due to the fragility of the pseudostratified respiratory epithelium. These lesions require a treatment approach similar to that used for an actual perforation.^74^

Unfortunately, due to the limited intraoperative visibility of the transcrestal approach, detecting membrane delamination with this technique is virtually impossible.

## 3 VASCULAR DAMAGE

Vascular injury, a risk associated only with the lateral approach, can lead to significant bleeding, primarily when the buccal osteotomy damages the intraosseous passage of the alveolo‐antral artery.^75^ This artery is an anastomosis between the infraorbital and posterosuperior alveolar arteries.^76^

**FIGURE 8:**
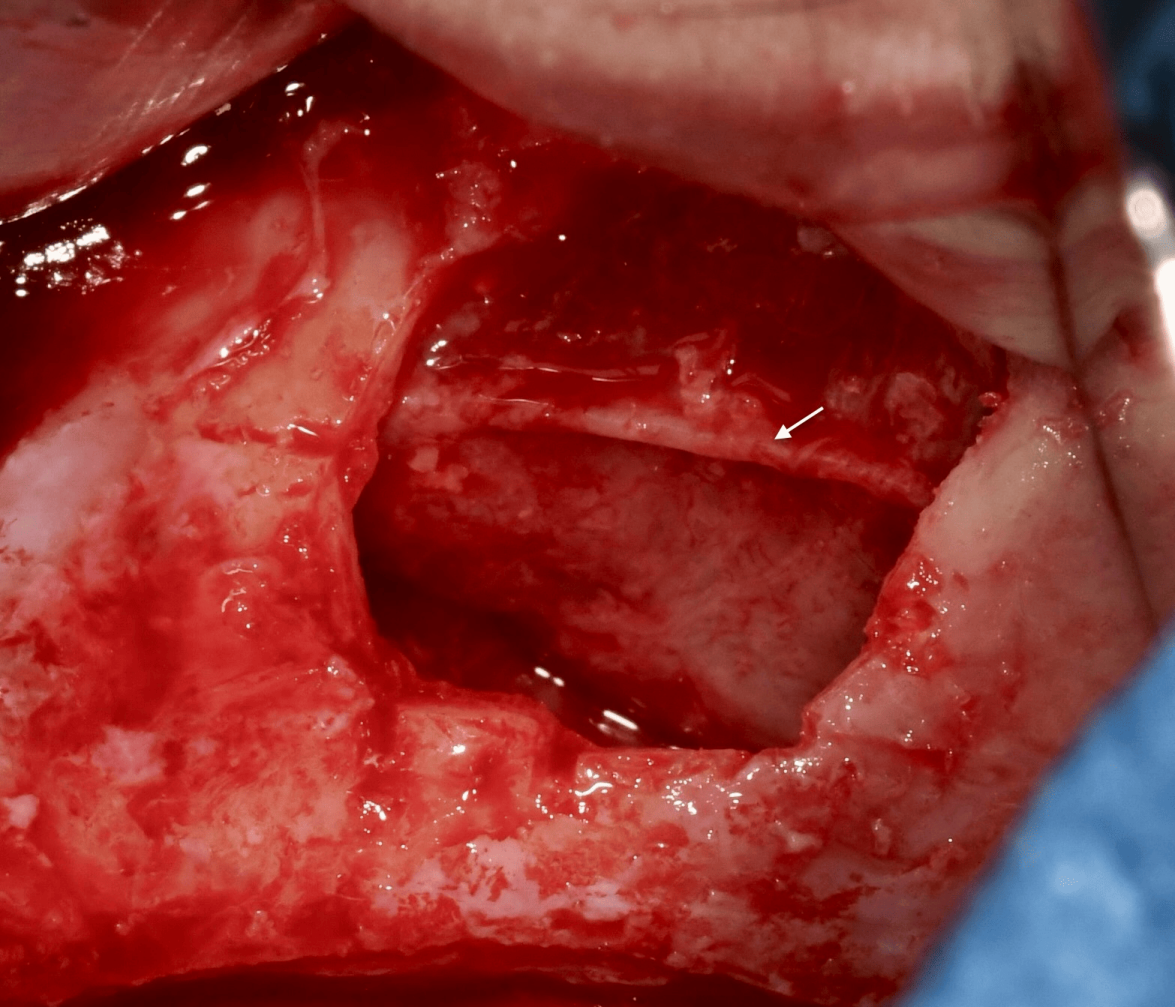
The AAA is stuck to the membrane (white arrow).

**FIGURE 9:**
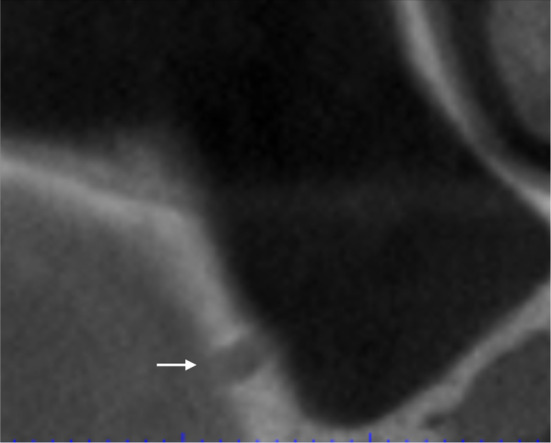
The intra osseous passage of the AAA (white arrow).

**FIGURE 10:**
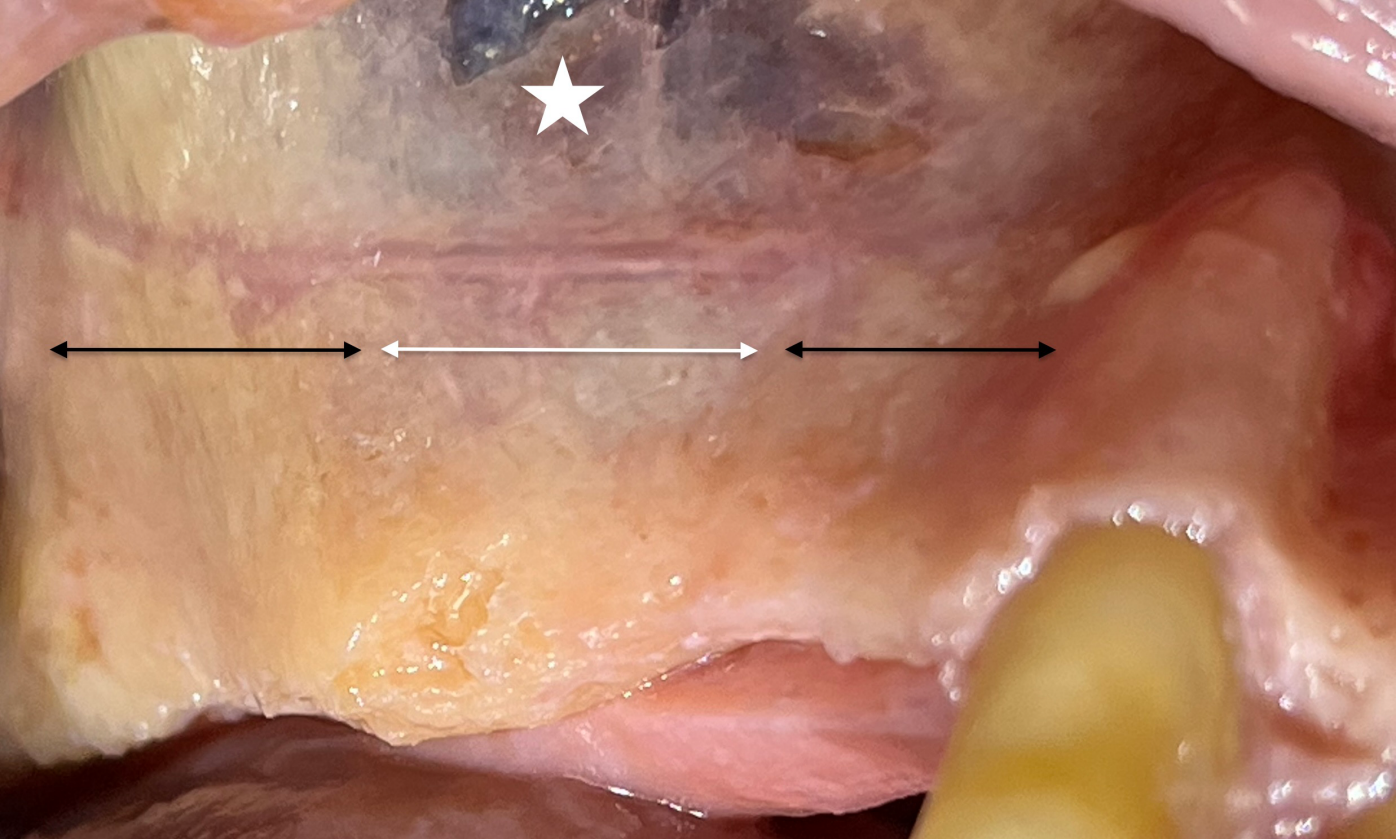
The artery has a intraosseous course distally and mesially (black double arrows) and an extraosseous course in the center (white double arrow) where the vestibular wall is extremely thin (white star).

Rosano and colleagues^76^ described three possible routes for the artery:

- An internal extraosseous pathway which is usually not detectable on cross‐sectional images because the vessel is stuck between the lateral wall and the sinus membrane (Figure 8). In this situation, there is no risk of bleeding during buccal antrostomy.
- A fully intraosseous path within the lateral wall of the maxillary sinus (Figure 9) is observed in 47% of cases, and this should be considered when planning the osteotomy in this area.^75^ The risk of bleeding should be taken into account.
- A buccal pathway between the lateral wall and the periosteum (Figure 10) that is not identifiable on coronal sections. This may be suspected when an intraosseous pathway is observed in the distal part within a relatively thick vestibular wall. This intraosseous pathway then disappears in presence of a thinner vestibular wall and reappears mesially within the thickness of a thicker wall. The vestibular extraosseous pathway is believed to result from the externalization of vessels due to pathological horizontal bone resorption following periodontal disease. Although this situation is uncommon, when suspected, great care must be taken when elevating the vestibular flap.

The frequency and severity of bleeding rise with the size of the artery. According to Testori et al., the probability of significant bleeding is 10% for a vessel diameter of 0.5–1 mm and increases to 57% for diameters larger than 2 mm.^13^, ^75^

### 3.1 Prevention and management of the vascular damage

Addressing intraoperative bleeding involves several approaches, including clamping the bleeding site,^77^ applying pressure with gauze treated with tranexamic acid,^46^ or using bone wax.^13^ Each technique carries its own risks, such as the potential for postoperative bleeding recurrence. Another effective method is diathermocoagulation with a bipolar electrosurgical unit, although this poses a risk for the integrity of the sinus membrane.^13^, ^75^ In cases of intraoperative bleeding, it may be beneficial to avoid suturing the distal incision as this can facilitate the expulsion of any clots.^78^

**FIGURE 11:**
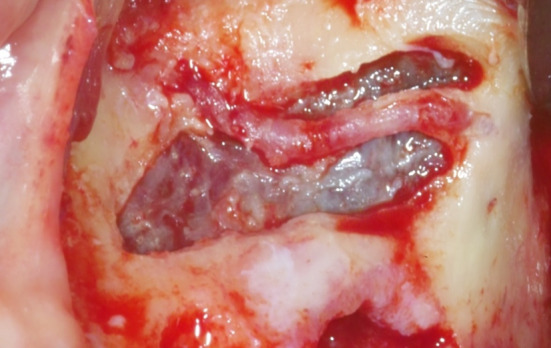
The artery is isolated.

**FIGURE 12:**
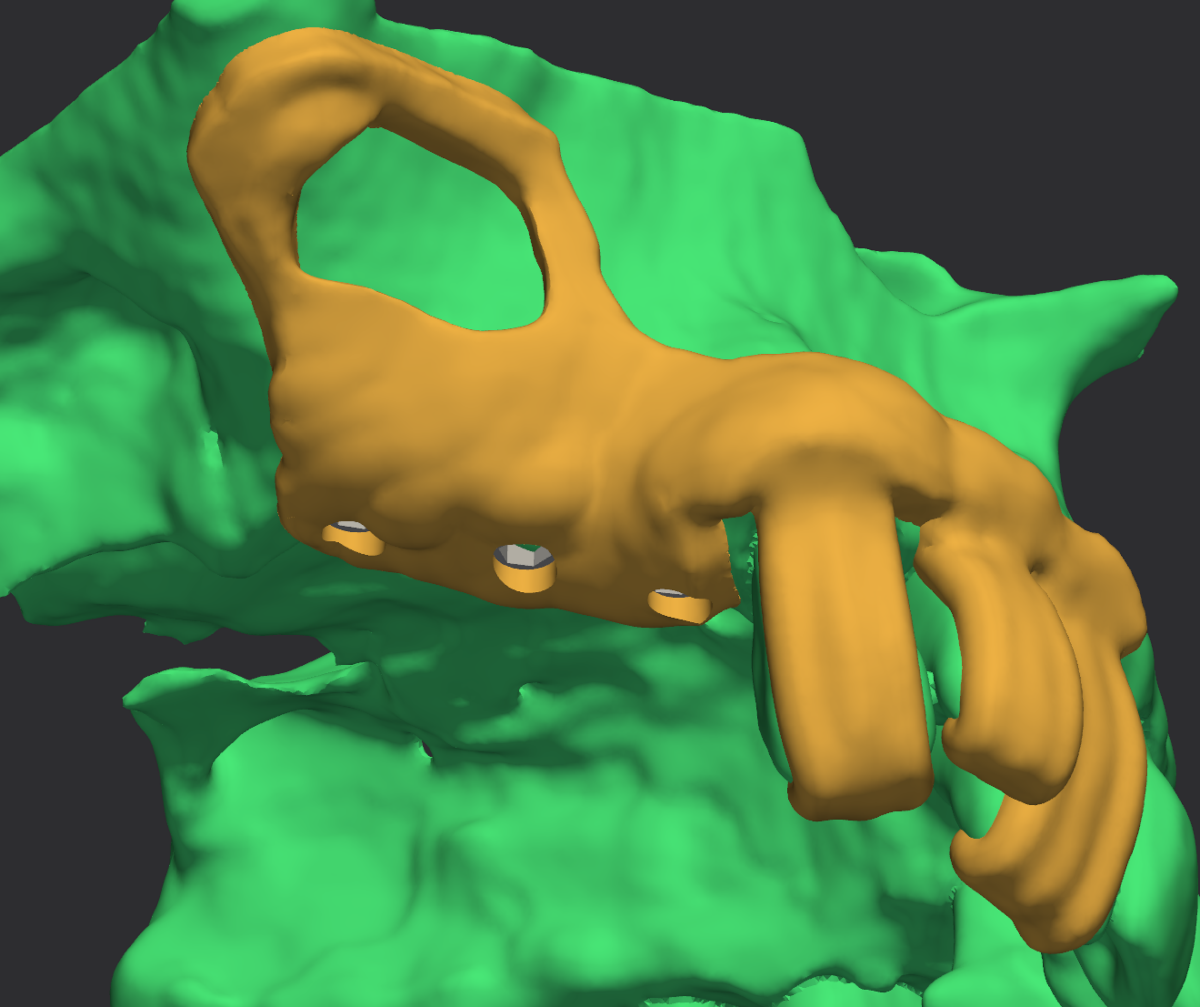
The 3D‐printed surgical guide.

The most effective approach to prevent bleeding involves careful isolation of the alveolo‐antral artery using piezoelectric surgical devices^42^, ^46^ (Figure 11). Additionally, reducing the size of the bony window and adjusting its positioning—either lower or higher when feasible—can also help minimize the risks of bleeding during the procedure. In this context, the use of 3D‐printed surgical guides derived from digital workflows could represent a significant aid for the clinician, providing real‐time guidance during antrostomy, and ensuring precise alignment of surgical steps with predetermined parameters established during the planning phase^79^ (Figure 12). This approach also reduces the need for intraoperative adjustments, resulting in shorter surgical times and improved overall workflow, ultimately leading to enhanced patient comfort and more predictable surgical outcomes.

## 4 NUMBNESS

The occurrence of neurosensory changes following sinus floor elevation with lateral approach, such as transient numbness on the operation side, is seldom highlighted but can impact patient comfort and recovery.

### 4.1 Risk factors

This kind of discomfort may be observed on the operated side after surgery, as a consequence of severing terminal branches of the infraorbital nerve during the mesial vertical releasing incision.^46^ Its frequency is related to bone atrophy degree: in more severe cases, the emergence of the infraorbital nerve is nearer to the operative area.

### 4.2 Prevention

**FIGURE 13:**
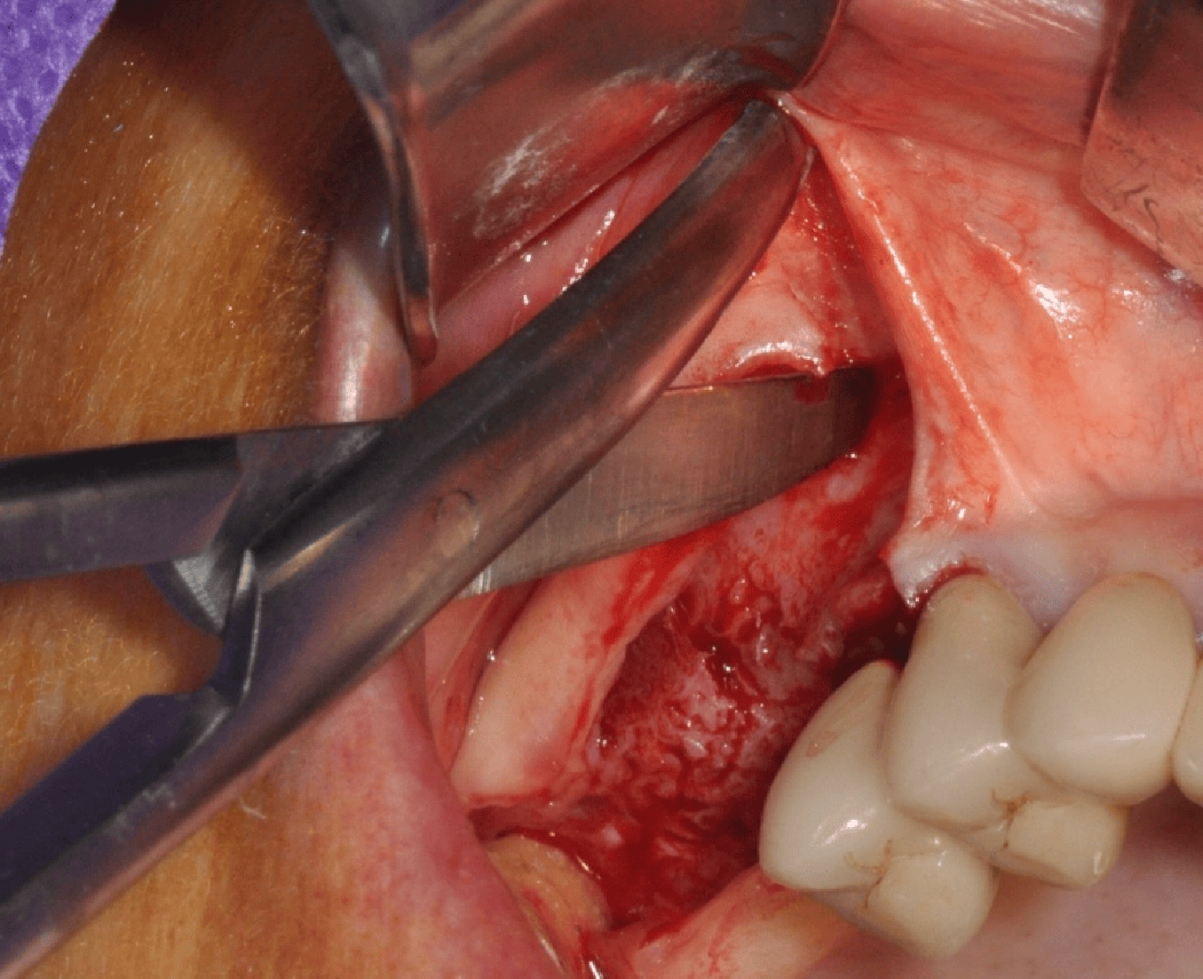
Metzenbaum scissors are inserted under the split thickness incision and opened to stretch the nerve branches without cutting them.

To prevent this issue, a nuanced approach involves making a shallower, partial thickness vertical releasing incision in the alveolar mucosa rather than full thickness. Subsequently, gently parting the incision edges with Metzenbaum scissors can stretch the nerve fibers without cutting them, thereby preserving sensory function (Figure 13). An alternative strategy consists of using a triangular flap without performing any mesial releasing incision.^78^

In the transcrestal approach, no extensive exposure of the lateral wall is required and a minimally invasive flap is performed, usually without releasing incisions. Therefore, postoperative numbness is not an issue when using this technique.

## 5 IMPLANT DISPLACEMENT

The risk of implant displacement into the sinus cavity requires careful attention when implants are inserted alongside grafting procedures. Achieving solid implant stability is paramount to avoid the risk of the implant migrating into the sinus or possibly moving into sensitive regions.^80^

### 5.1 Risk factors and prevention

Two primary factors that can contribute to implant displacement are: firstly, the presence of a residual bone crest with extremely low quality, which can be addressed by utilizing the osseodensification technique to improve bone density.^81^ Secondly, very limited bone height below the sinus (<3 mm) during simultaneous implant placement. To address this, drilling should be undersized, and tapered implants should be used, although this approach may potentially fracture the vestibular wall due to high pressure transmitted to the bone during implant insertion. To mitigate this risk, the surgical window can be adjusted apically by 8–10 mm, providing a more secure setting for the implant.^46^, ^53^ Additionally, using cover screws larger than the implant diameter can also be considered.

## 6 POOR GRAFT ADAPTATION

**FIGURE 14:**
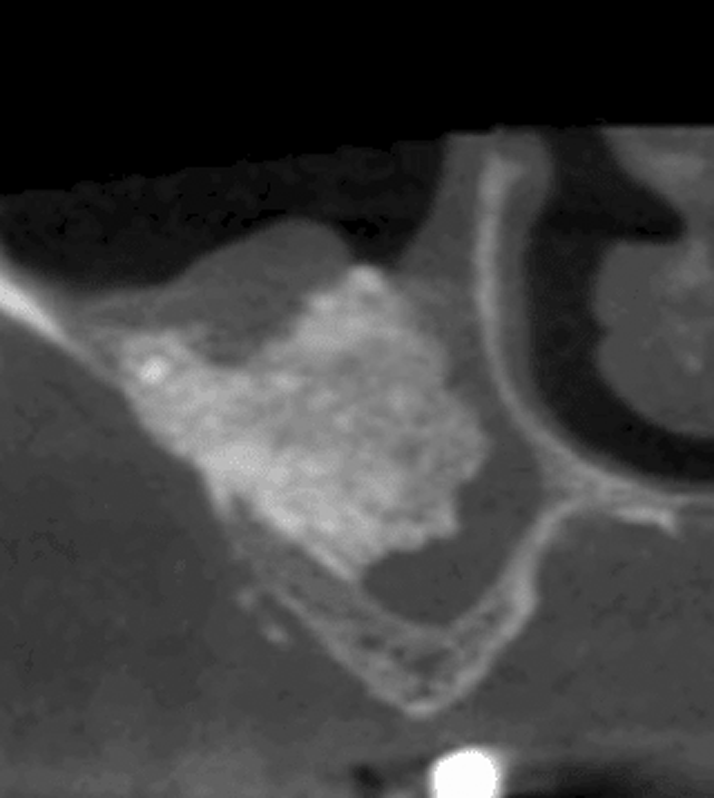
The grafting material is not in contact with the medial wall.

**FIGURE 15:**
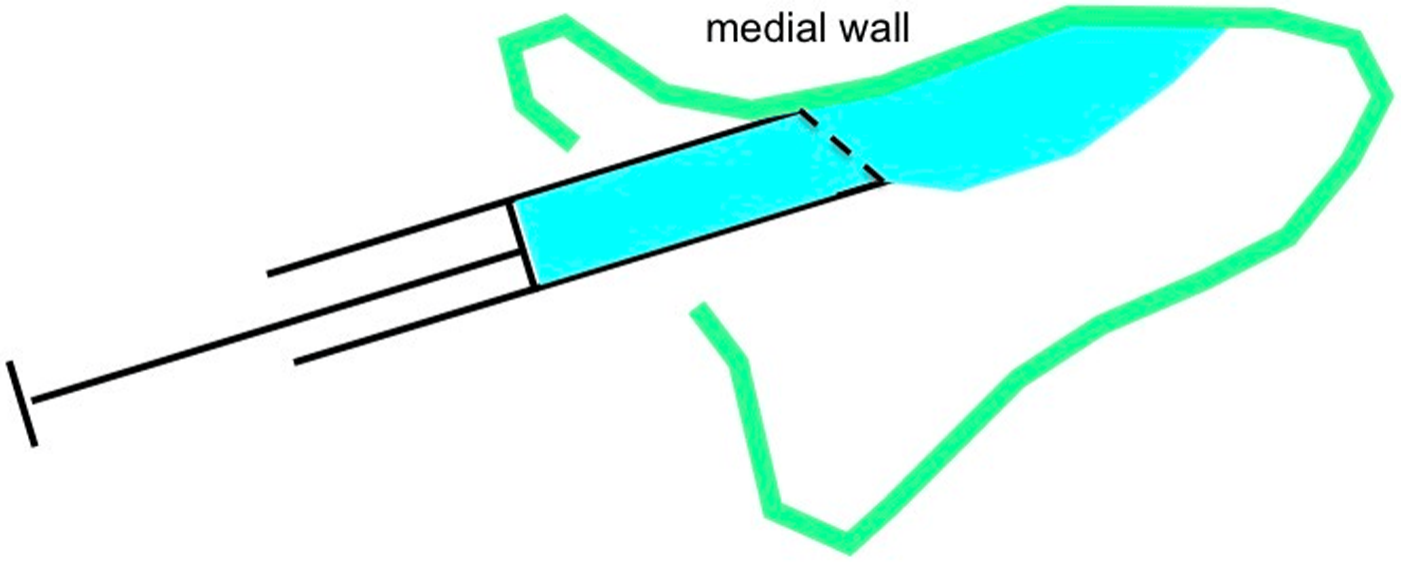
The grafting technique using a beveled (dotted line) insulin syringe to place the graft material in direct contact with the medial wall.

To ensure optimal bone quality, it is essential to achieve maximum contact between the graft and the surrounding native bone.^46^ This necessitates avoiding gaps (Figure 14) by ensuring that the grafting material directly contacts both the medial and anterior walls. An insulin syringe with a beveled tip is employed for this purpose in the lateral sinus augmentation. The syringe, loaded with grafting material, is inserted into the sinus cavity in a backward and inward direction until it reaches the wall to be grafted,^46^ aligning the bevel towards the wall (Figure 15). The material is then injected. It is important that the dimensions of the buccal bone window are appropriate to allow for the execution of this technique, while remaining as small as possible.^82^

Even in the transcrestal approach, it is necessary to ensure that the graft comes into contact with the sinus walls to optimize bone formation.^11^, ^83^ The clinician must take care to angle the tip of the biomaterial syringe in various directions (buccal, palatal, mesial, and distal) if using injectable material, to fill the sub‐antral space as evenly as possible. The same outcome should also be achieved when using particulate graft. Once the biomaterial is placed beneath the membrane, the granules can be gently pushed in various directions using small compactors inserted through the crestal antrostomy. In transcrestal sinus augmentation, achieving a uniform distribution of the grafting material can be reliably obtained when the sinus cavity is narrow in bucco‐palatal direction.^11^

## 7 BENIGN PAROXYSMAL POSITIONAL VERTIGO

Benign paroxysmal positional vertigo (BPPV) is an uncommon complication following transcrestal sinus floor elevation performed by using osteotomes.^84^, ^85^ BPPV can occur due to the displacement of inner ear crystals (otoconia) into the semicircular canals, leading to episodes of vertigo triggered by head movements. The etiology of BPPV is likely associated with the surgical trauma induced by osteotomes and a surgical hammer during bone malleting and condensation to create the crestal antrostomy, leading to the displacement of otoconia. Prevention strategies include minimizing excessive force or rapid movements during the procedure and ensuring careful manipulation to reduce the risk of otoconia displacement. The use of the magnetic mallet instead of the manual surgical mallet could be an effective strategy in preventing BPPV, as there have been no reported cases of BPPV in the literature with the use of this device. However, given the low frequency of this complication and the limited number of studies conducted on this topic, further data are needed to confirm the preventive action of this technology on BPPV. Treatment options for BPPV include canalith repositioning maneuvers (e.g., Epley maneuver) to guide the displaced otoconia back to their original position within the inner ear, alleviating symptoms of vertigo. Additionally, patients may benefit from vestibular rehabilitation exercises to improve balance and reduce the frequency of vertigo episodes following transcrestal sinus floor elevation.

## 8 CONCLUSIONS

Intraoperative complications during sinus augmentation procedures, particularly sinus membrane perforation, pose significant challenges and necessitate careful consideration of various risk factors and preventive strategies. The prevalence of membrane perforation varies between lateral and transcrestal approaches, with distinct implications for surgical techniques and anatomical factors. Risk factors such as antrostomy technique, anatomy (including membrane thickness, gingival phenotype, sinus width, and septa), patient‐related factors (such as surgical access and smoking), and specific procedural aspects must be thoroughly evaluated with a meticulous presurgical planning to minimize the occurrence of intraoperative complications. The implementation of advanced techniques and technologies, to be used both in the planning and in the operational phase, can contribute to enhance surgical precision, ultimately improving patient comfort and treatment predictability.

It is important to consider that a close collaboration with the otorhinolaryngologist is necessary in managing patients who have experienced intraoperative complications, with the aim of a rapid and effective multidisciplinary resolution of the problem.

## CONFLICT OF INTEREST STATEMENT

The authors declare no conflicts of interest.

## Data Availability

Not applicable.
